# Targeting Tumor Endothelial Cells by EGCG Using Specific Liposome Delivery System Inhibits Vascular Inflammation and Thrombosis

**DOI:** 10.1002/cam4.70462

**Published:** 2024-12-04

**Authors:** Zi Jia, Nako Maishi, Hideki Takekawa, Aya Yanagawa Matsuda, Taisei Nakade, Takashi Nakamura, Hideyoshi Harashima, Yasuhiro Hida, Kyoko Hida

**Affiliations:** ^1^ Vascular Biology and Molecular Pathology Hokkaido University Graduate School of Dental Medicine Sapporo Japan; ^2^ Faculty of Pharmaceutical Sciences Hokkaido University Sapporo Japan; ^3^ Advanced Robotic and Endoscopic Surgery School of Medicine, Fujita Health University Toyoake Japan

**Keywords:** EGCG, ROS, tumor endothelial cell, tumor inflammation, tumor thrombosis

## Abstract

**Background:**

Inflammation is one of the hallmarks of cancer and is associated with tumor growth. Tumor endothelial cells (TECs) demonstrate inflamed phenotypes. Endothelial inflammation initiates thrombus formation, which is the second cause of cancer‐related deaths. Epigallocatechin‐3‐O‐gallate (EGCG), a natural compound in green tea, has demonstrated an anti‐inflammatory effect. However, the tumor progression inhibition effect of EGCG by targeting TEC inflammation remains unclear. This study addresses the anti‐tumor effect of EGCG, especially its anti‐inflammatory role in TECs.

**Methods:**

In vitro, the effect of EGCG on TECs were studied using real‐time quantitative PCR and immunofluoresence to analyza gene and protein expression. In vivo, a cyclic RGD liposome delivery system (MEND) was employed to efficiently deliver EGCG to TECs in tumor‐bearing mice.

**Results:**

In vitro, EGCG significantly reduces inflammatory cytokine expression, including tumor necrosis factor‐α, interleukin‐6, IL‐8, and IL‐1β through NF‐κB signaling inhibition. Additionally, von Willebrand factor reduction in TECs, which is involved in platelet adhesion and thrombosis formation, was analyzed. Our results revealed that EGCG‐MEND significantly inhibited TEC inflammation and thrombus formation in tumors. Additionally, EGCG‐MEND improved tumor immunity by reducing programmed death‐ligand 1 expression and promoting high endothelial venule formation by recruiting CD8^+^ T cells.

**Conclusion:**

Our results indicate the anti‐tumor potential of EGCG‐MEND in normalizing the inflammatory immune microenvironment and inhibiting thrombosis by targeting TEC.

## Graphical abstract

In this study, we demonstrated that EGCG effectively inhibited the expression of inflammatory cytokines such as TNF‐α and IL‐6 in TEC. In addition, we have developed a cyclic RGD peptide‐modified liposome delivery system (MEND) that specifically targets TEC. After injection into mice, we found that EGCG‐MEND effectively inhibited perivascular inflammation and thrombosis. Therefore, targeting TEC inflammation by EGCG delivery system may be beneficial for anti‐thrombosis strategy, making it a promising approach for cancer therapy.

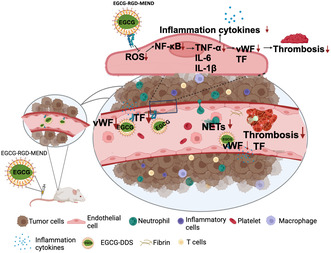

**Abbreviations**
cRGD: cyclo (Arg‐Gly‐Asp‐D‐Phe‐Lys)
DDS: drug delivery system
DHE: dihydroethidium
EGCG: epigallocatechin‐3‐O‐gallate
IHC: immunohistochemistry
LPS: lipopolysaccharide
MEND: multi‐enveloped nanodevices
MPO: myeloperoxidase
MVD: microvessel density
NETs: neutrophil extracellular traps
PD‐1: programmed cell death 1
PD‐L1: programmed death‐ligand 1
ROS: reactive oxygen species
TEC: tumor endothelial cells
TLR: toll‐like receptors
TNF‐α: tumor necrosis factor‐α
VEGF: vascular endothelial growth factor

## 1 Introduction

Angiogenesis is necessary for tumor progression and metastasis [1]. Tumor cells secrete vascular endothelial growth factor (VEGF) to induce angiogenesis and provide oxygen and nutrients for tumor development [2]. Angiogenesis inhibitors that generally block VEGF signaling exert their antitumor effects by disrupting tumor blood supply [3] and normalizing vascular structural abnormalities caused by excess VEGF, improving drug delivery and immune cell mobilization [4]. These improve outcomes in patients with cancer when used in combination with anticancer agents and/or immune checkpoint inhibitors [5]. However, VEGF inhibitors affect normal blood vessels because normal endothelial cells (NECs) also require VEGF [6]. Tumor endothelial cells (TECs) are different from NECs in many aspects, such as gene expression profile [7], proangiogenic properties [8], and drug sensitivity [9]. Because anti‐VEGF therapy has various side effects [10], a novel approach to target TECs independently of VEGF signaling is necessary.

In recent years, tumor‐associated chronic inflammation was found to promote immunosuppression of the tumor microenvironment and tumor progression [11]. Recent studies have demonstrated that TECs augment proinflammatory cytokines (tumor necrosis factor‐α [TNF‐α], interleukin‐6 [IL‐6], IL‐8, etc.) of cancer cells, which in turn activates nuclear factor kappa‐light‐chain‐enhancer of activated B cells (NF‐κB) and signal transducer and activator of transcription 3 (STAT3) signaling in cancer cells, and increase invasiveness in vitro [12]. Our previous studies have revealed the involvement of LOX‐1 in TECs in neutrophil migration via C‐C Motif Chemokine Ligand 2 (CCL2) signaling. The binding of oxidized LDL (ox‐LDL) to LOX‐1 further increases tumor metastasis [13]. Additionally, we have reported that TECs upregulated biglycan that modulates inflammatory responses through toll‐like receptors (TLR) [14]. Additionally, TECs in highly metastatic tumors demonstrate increased expression levels of inflammation‐related genes, including IL‐6, S100 calcium binding protein A (S100A), and cyclooxygenase‐2 (COX‐2) [15, 16]. Based on inflammation‐related gene expression on TECs, investigating the specific targeting of TEC through inflammation might be worthwhile. Leukocytes often aggregate around tumor blood vessels [17] and stimulate angiogenesis and tumor metastasis [18]. Numerous studies have confirmed that thrombosis is a common complication in patients with cancer and is the second leading cause of cancer‐related deaths [19]. Inflammatory cytokines, such as TNF‐α, IL‐6, and IL‐8, promote the procoagulant phenotype of ECs and increase platelet activation [20], and TNF‐α and IL‐1β induce tissue factor (TF) and von Willebrand factor (vWF) expression on ECs, thereby contributing to platelet aggregation and thrombosis [21]. This interrelationship between inflammation and thrombosis emphasizes the importance of investigating both aspects in patients with cancer. In particular, in‐depth research is warranted on their interactions and functional mechanisms in tumor microenvironment formation, alongside the potential effect of thrombosis during cancer treatment. This study aims to determine the specific role of TEC inflammation and thrombosis in tumor progression, and thus provide a basis for new cancer treatment strategies.

Epigallocatechin‐3‐O‐gallate (EGCG) is the most abundant polyphenolic compound in green tea [22]. EGCG is a natural antioxidant and anti‐inflammatory agent that has shown potenrial in the treatment of cancer and multiple diseases [23, 24, 25]. Its antioxidant effect is mainly achieved by scavenging free radicals, inhibiting lipid peroxidation, and increasing endogenous antioxidant enzyme activities, thereby reducing oxidative stress damage to cells [26]. Additionally, EGCG inhibits the release of proinflammatory factors by regulating multiple inflammatory signaling pathways, including NF‐κB and mitogen‐activated protein kinase (MAPK) pathways, thereby exhibiting anti‐inflammatory effects [27]. Previously, we reported that EGCG inhibited TEC proliferation and migration but not NEC [9]. However, the anti‐inflammatory effect of EGCG on TECs is currently unclear. In this study, we expected EGCG to achieve anti‐inflammatory and antithrombosis effects.

EGCG belongs to the Biopharmaceutics Classification System III class drug, characterized by high solubility but low permeability, and exhibits lower absorption rates of < 10% and lower oral bioavailability [28]. This is because TEC inflammation is involved in promoting tumor progression [12]. Therefore, targeting TECs is crucial for inhibiting inflammatory tumor microenvironment and inflammation‐related thrombosis, and finally, achieving anti‐tumor therapeutic efficacy. Therefore, delivering EGCG specifically to TECs is crucial to overcome the limitations of EGCG and effectively target TECs for anti‐inflammation purposes. We previously established a drug delivery system (DDS) with lipid nanoparticles of multi‐enveloped nanodevices (MEND) that achieved specific drug delivery to TECs [29]. The binding of cyclo (Arg‐Gly‐Asp‐D‐Phe‐Lys) (cRGD) to MEND [29] resulted in the specific delivery of its contents to TECs, as cRGD binds to α V β 3 integrin, a receptor highly expressed on TEC [30]. In earlier studies, we revealed the therapeutic efficacy of MEND for its antiangiogenesis and antitumor effects, by silencing the vegfr2 [31] or biglycan [32] genes in TECs using mouse tumor models. We further demonstrate that the use of the RGD‐MEND system to deliver EGCG effectively inhibits inflammation and thrombus formation in TECs, thereby presenting a novel approach to cancer treatment, indicating the high and specific efficacy of the MEND system in targeting TECs.

## 2 Materials and Methods

### 2.1 Cell Lines and Culture Conditions

MS1 cells, murine immortalized islet‐derived NECs, were obtained from American Type Culture Collection (Manassas, VA, USA) and cultured in Dulbecco's minimum essential medium (DMEM, Sigma–Aldrich, St. Louis, MO, USA) supplemented with 10% heat‐inactivated fetal bovine serum (FBS) and 1% penicillin/streptomycin antibiotics (Sigma–Aldrich). MS1 cells, which are immortalized mouse NECs, maintain the expression of common endothelial markers and preserve key endothelial characteristics [33]. Lewis lung carcinoma (LLC) cells were purchased from RIKEN Cell Bank (Tsukuba, Ibaraki, Japan), and CT26 cells were purchased from American Type Culture Collection (Manassas, VA, USA) cultured in DMEM and RPMI‐1640 (#R8758, Sigma‐Aldrich) supplemented with 10% FBS and 1% penicillin/streptomycin, respectively. Cells were cultured at 37°C in a humidified atmosphere containing 5% CO_2_. All cells were free of mycoplasma contamination as confirmed by polymerase chain reaction (PCR).

### 2.2 Isolation and Culture of TECs

We utilized endothelial cells isolated from LLC tumors as a control model for TECs, as LLC tumor growth has induced progressively increasing peripheral inflammation [34]. TECs from LLC tumors were isolated as previously described with modifications [35, 36]. Briefly, 1 × 10^6^ LLC cells were injected subcutaneously into C57BL/6JJcl mice (female, 7 weeks old, CLEA Japan, Tokyo, Japan). Tumor tissues grown in the mice were resected and minced, and TECs were isolated using a magnetic cell sorting system (Miltenyi Biotec, Tokyo, Japan) with anti‐mouse CD31 microbeads following the manufacturer's protocol. To enhance the purity of TEC, CD45−/CD31+ cells were further sorted using a FACS Aria II (BD Biosciences). Isolated cells were cultured in EGM‐2MV medium (Lonza, Basel, Switzerland) supplemented with 15% FBS at 37°C in a humidified atmosphere with 5% CO_2_. After seeding in a culture plate and reaching 80% confluence, TECs were subjected to a second round of FACS for purification, and purified cells were cultured in an EGM‐2MV culture medium.

### 2.3 Cell Proliferation Assays

Cell viability was measured by the MTS assay using the CellTiter 96 Aqueous One Solution Cell Proliferation Assay Kit (Promega, Madison, WI). TECs and NECs were seeded into 96‐well plates (1000 cells/well) and pretreated with EGCG (Sigma, E4143) at various concentrations (5–100 μM) or vehicle (dimethyl sulfoxide [DMSO]) for 48 h. After incubation, cell viability was determined by measuring absorbance at 490 nm (OD490).

### 2.4 EGCG Treatment and Stimulation of Cells

LLC‐ECs were cultured in EGM‐2MV overnight before treatment with 50 or 100 μM EGCG. EGCG exhibits possess anti‐inflammatory and anti‐tumor properties, but its efficacy varies by dose [37]. We analyzed multiple concentrations (data not shown) and revealed that 50 or 100 μM of EGCG improved therapeutic outcomes, such as inhibiting TEC inflammation and tumor progression. This enables us to optimize the use of EGCG in targeting TECs. Therefore LLC‐ECs were cultured in EGM‐2MV overnight before treatment with 50 or 100 μM of EGCG. After 6 h of incubation with EGCG, RNA was isolated. As a control, an equal volume of DMSO was used in the NT group to account for solvent effects. MS1 cells were pretreated with LPS (500 ng/mL, InvivoGen, tlrl‐3pelps) for 6 h and then treated with 50 μM EGCG for 6 h before RNA extraction. LLC‐ECs were treated with the NF‐κB inhibitor BAY11‐7082 (10 μM, Calbiochem) for 6 h, and RNA was isolated.

### 2.5 RNA Isolation and Real‐Time Quantitative PCR (qRT‐PCR)

RNA was isolated using the ReliaPrep RNA Cell Miniprep System (Promega) according to the manufacturer's instructions. The cDNA was synthesized using ReverTra‐Plus (Toyobo Co., Japan). qRT‐PCR was conducted using KAPA SYBR Fast qPCR Kit (Nippon Genetics). mRNA expression levels were normalized to Gapdh and analyzed using the delta‐delta‐Ct method. The primers used are listed in Table S1.

### 2.6 Dihydroethidium (DHE) Staining and Immunocytochemistry

The superoxide‐sensitive dye DHE (Sigma‐Aldrich) was used to detect intracellular reactive oxygen species (ROS). After pretreatment with the indicated dose of EGCG for 1 h, cells were incubated with DHE (10 μM) for 30 min at 37°C in the dark. The cells were fixed in 4% paraformaldehyde and stained with phospho‐NF‐κB p65 antibody (Ser536) (93H1) (3033S, Cell Signaling), followed by Alexa Fluor 488‐conjugated goat anti‐rabbit IgG (A‐11008, Invitrogen). Cells were counterstained with 4,6‐diamidino‐2‐phenylindole (DAPI; Dojin Chemical, Kumamoto, Japan), and images were obtained using a BZ‐X810 microscope equipped with BZ‐X800 Analyzer software (Keyence Corporation, Itasca, IL, USA).

### 2.7 Mouse Tumor Model

All animal experiments were approved by the Animal Research Authority of Hokkaido University. C57BL/6JJcl mice (female, 7 weeks old, 17–20 g) were purchased from CLEA (Japan), were used due to their propensity to produce higher levels of antibodies and stronger inflammatory responses [38]. CT26 cells (8 × 10^5^) were subcutaneously implanted into the right flanks of mice. Treatment started when the tumor volume reached 100 mm^3^. Mice were randomly assigned to two groups (*n* = 4): the EGCG‐MEND (10 mg/kg) group and the control group (Ctr‐MEND, the same concentration of MEND treatment as the experimental group). Ctr‐MEND or EGCG‐MEND was administered three times weekly for a total of five administrations by intravenous injections via a tail vein. Tumor volumes were measured with a caliper every 3 days and calculated using the following formula: volume = largest dimension × smallest dimension^2^ × 0.5.

### 2.8 Immunohistochemistry (IHC)

Detailed descriptions of the methods are provided in Appendix S1.

### 2.9 Evaluation of IHC Staining

Detailed descriptions of the methods are provided in Appendix S1.

### 2.10 Preparation of EGCG‐RGD‐MEND

RGD‐MEND was prepared as described previously [39, 40, 41]. For MEND preparation, lipid components including 2100 nmol YSK05, 900 nmol cholesterol, and 30 nmol PEG2k‐DMG were dissolved in 90% aqueous tertiary butanol (total volume of 400 μL). Subsequently, 10 mg/mL EGCG (60.6 μL) was added to the lipid solution under vortexing. The mixture was added to the 20 mM citrate buffer under vortexing. Furthermore, 3.5 mL of PBS was added to the mixture under vortexing. t‐BuOH was removed for ultrafiltration. For RGD modification, 0.06 mM of RGD–PEG solution was incubated with YSK‐MEND at 60°C. A Zetasizer Nano ZS ZEN3600 instrument was used for characterization, with a measured diameter of 236.5 nm, a PDI of 0.073, and an ζ‐potential (mV) of −10.1 (Table S2). The loading efficiency of EGCG was evaluated at Abs of 275 nm and was approximately 90%. Transmission electron microscopy verified the spherical morphology of the nanoparticles [42].

### 2.11 Statistical Analysis

Data are expressed as means ± standard deviations of three independent experiments performed in triplicates. Student's *t*‐test was used for comparison between two groups, and a one‐way analysis of variance test was used among three or more groups. *p* < 0.05 was considered significant.

## 3 Results

### 3.1 EGCG Exerts Antiproliferative and Anti‐Inflammatory Effects on TECs

**FIGURE 1:**
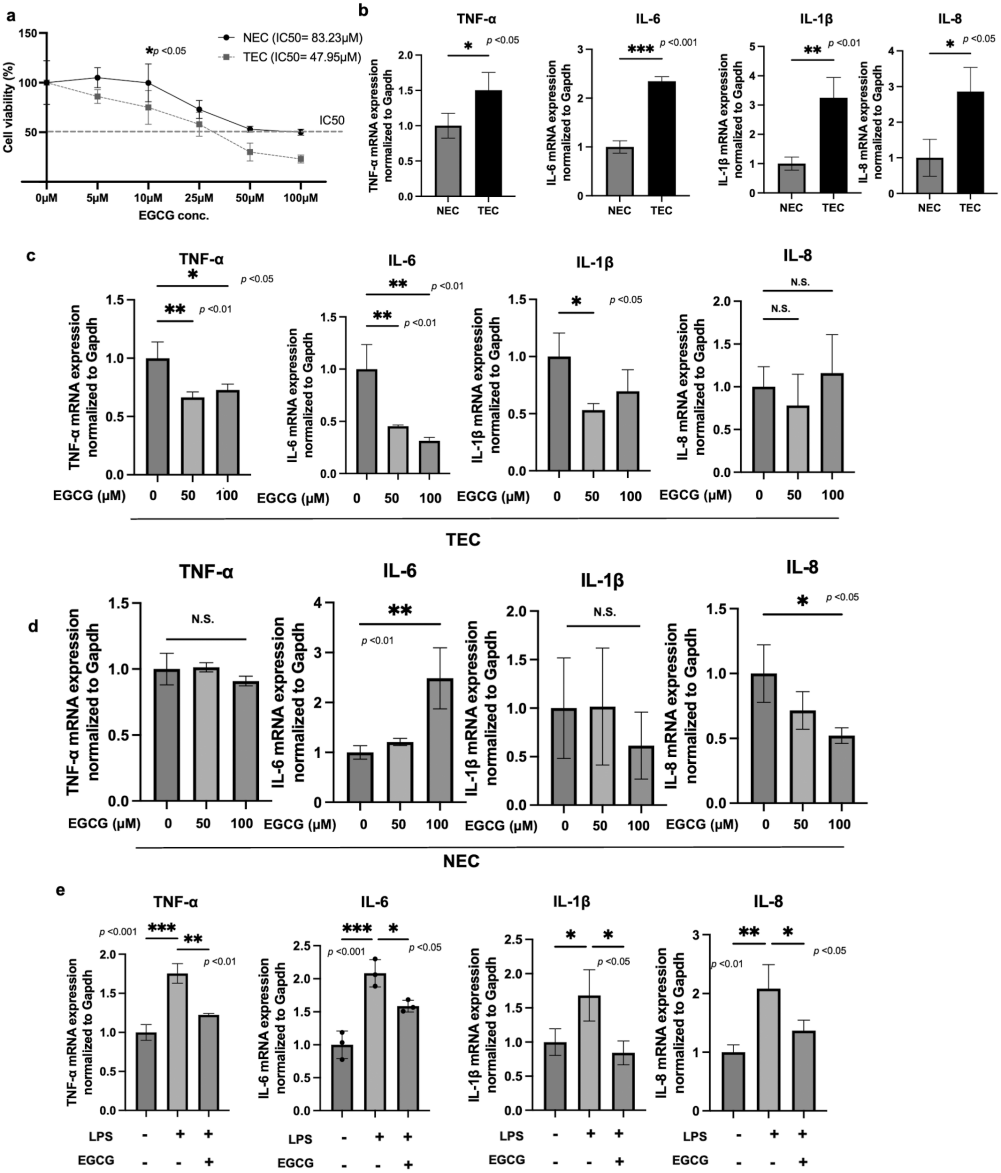
EGCG exerts antiproliferative and anti‐inflammatory effects on TECs. (a) TECs and NECs were incubated in the EGCG‐containing media for 48 h, and their viability was assessed by the MTS assay. (b) The mRNA expressions of TNF‐α, IL‐6, IL‐1β, and IL‐8 in TECs and NECs were determined by RT‐qPCR. (c, d) the mRNA expression in TECs and NECs was determined by RT‐qPCR after treatment with the indicated dose of EGCG for 6 h. (e) NECs were stimulated with LPS and then treated with EGCG, and the expression levels of inflammation‐related genes (TNF‐α, IL‐6, and IL‐1β) were demonstrated by RT‐qPCR. Results presented as mean ± SD; *p < 0.05, **p < 0.01, ***p < 0.001; one‐way ANOVA (a–e) and Student's *t*‐test (b) were used.

To evaluate the effect of EGCG on TECs and NECs LLC‐ECs were used as TECs isolated from LLC tumors grown in mice and MS1 cells as NECs. First, the antiproliferative effect of EGCG on ECs was analyzed. The IC50 value of EGCG was calculated to be 47.95 μM and 83.23 μM for TECs and NECs, respectively. TECs were more sensitive to EGCG than NECs (*p < 0.05) (Figure 1a), which is consistent with our previous report [9]. Then, the expression of inflammatory cytokines between TECs and NECs was compared, and a significant upregulation of these genes was found in TECs (Figure 1b). To determine whether EGCG exerts anti‐inflammatory effects on ECs as reported, qRT‐PCR was performed after EGCG treatment. The gene expression levels of TNF‐α, IL‐6, and L‐1β decreased in TECs after EGCG treatment (Figure 1c), whereas no significant changes in NECs were found (Figure 1d). The difference in the expression of IL‐8 between TECs and NECs was not significant, and to investigate the changes in the expression of inflammatory factors in TECs, the expression levels of TNF‐α, IL‐6, and IL‐1β genes were assessed. To determine whether EGCG exerts anti‐inflammatory effects on inflammatory changes in response to inflammatory stimuli, NECs were treated with EGCG after lipopolysaccharide (LPS) stimulation. EGCG reduced the gene expression levels of TNF‐α, IL‐6, IL‐1β and IL‐8 in LPS‐stimulated NECs (Figure 1e). These results suggested the anti‐inflammatory effects of EGCG on inflammatory ECs including TECs.

### 3.2 EGCG Scavenging ROS in TECs Achieves Anti‐Inflammatory Effects

**FIGURE 2:**
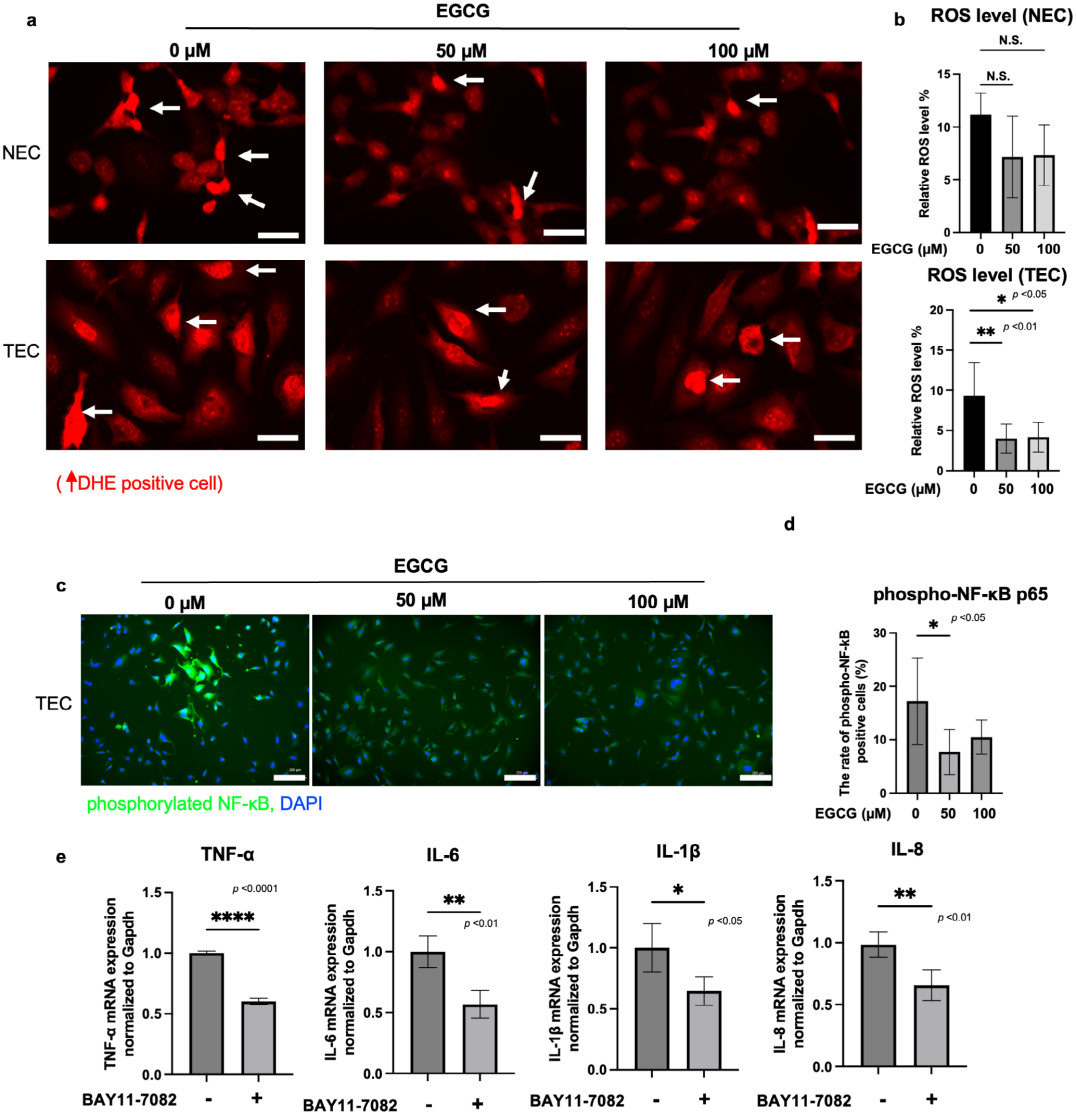
EGCG scavenging ROS in TECs to achieve anti‐inflammatory effects. (a) After EGCG treatment of TECs and NECs for 1 h, ROS level (red) was determined by DHE staining. Scale bar = 100 μM. (b) DHE‐positive cells were counted per field, *n* = 5 fields. (c) After treatment with the indicated dose of EGCG in TECs, the p‐NF‐κB (green) was evaluated by immunocytochemistry. DAPI (blue) marks the nucleus. Scale bar = 200 μM (d) the nuclear/cytoplasmic ratio of p‐NF‐κB signals was quantified using ImageJ, (e) after treatment with the NF‐κB inhibitor (BAY11‐7082), TNF‐α, IL‐6, and IL‐1β mRNA expression levels were evaluated using RT‐PCR (*p < 0.05, **p < 0.01, ****p < 0.0001, N.S., not significant); *n* = 5 per group analyzed by unpaired Student's *t*‐test.

EGCG was demonstrated to exert its anti‐inflammatory effects by scavenging reactive oxygen species (ROS) [43]. Previously, we have suggested ROS accumulation in tumor blood vessels in vivo [3]. In DHE staining, ROS signals between NECs and TECs were no difference in the in vitro culture as we reported previously [44]. However, EGCG treatment reduced ROS levels by > 50% in TECs compared to the untreated group (Figure 2a,b). In cancer, growth factors and cytokines (e.g., TNF‐α) were reported to be one of the ROS‐generating factors [45]. EGCG may have greater effects on TECs than on NECs because of expression levels of cytokines. These results suggested that EGCG scavenges ROS in TECs while acting as an anti‐inflammatory agent and antioxidant in TECs. Because ROS was reported to activate NF‐κB signaling and upregulate inflammation‐related genes including TNF‐α and IL‐1β [46], immunocytochemistry was performed to analyze NF‐κB phosphorylation in TECs with or without EGCG treatment. TECs with NF‐κB phosphorylation were detected; expectedly, it was inhibited by EGCG treatment (*p < 0.05) (Figure 2c,d). Moreover, the NF‐κB inhibitor BAY11‐7082 decreased the gene expression levels of TNF‐α, IL‐1β, IL‐6 and IL‐8 in TECs (Figure 2e). These results suggest that EGCG, which acts as an antioxidant by scavenging ROS, inhibits NF‐κB activity, thereby downregulating inflammation‐related genes in TECs.

### 3.3 EGCG‐MEND Possesses Anti‐Angiogenic Effects

**FIGURE 3:**
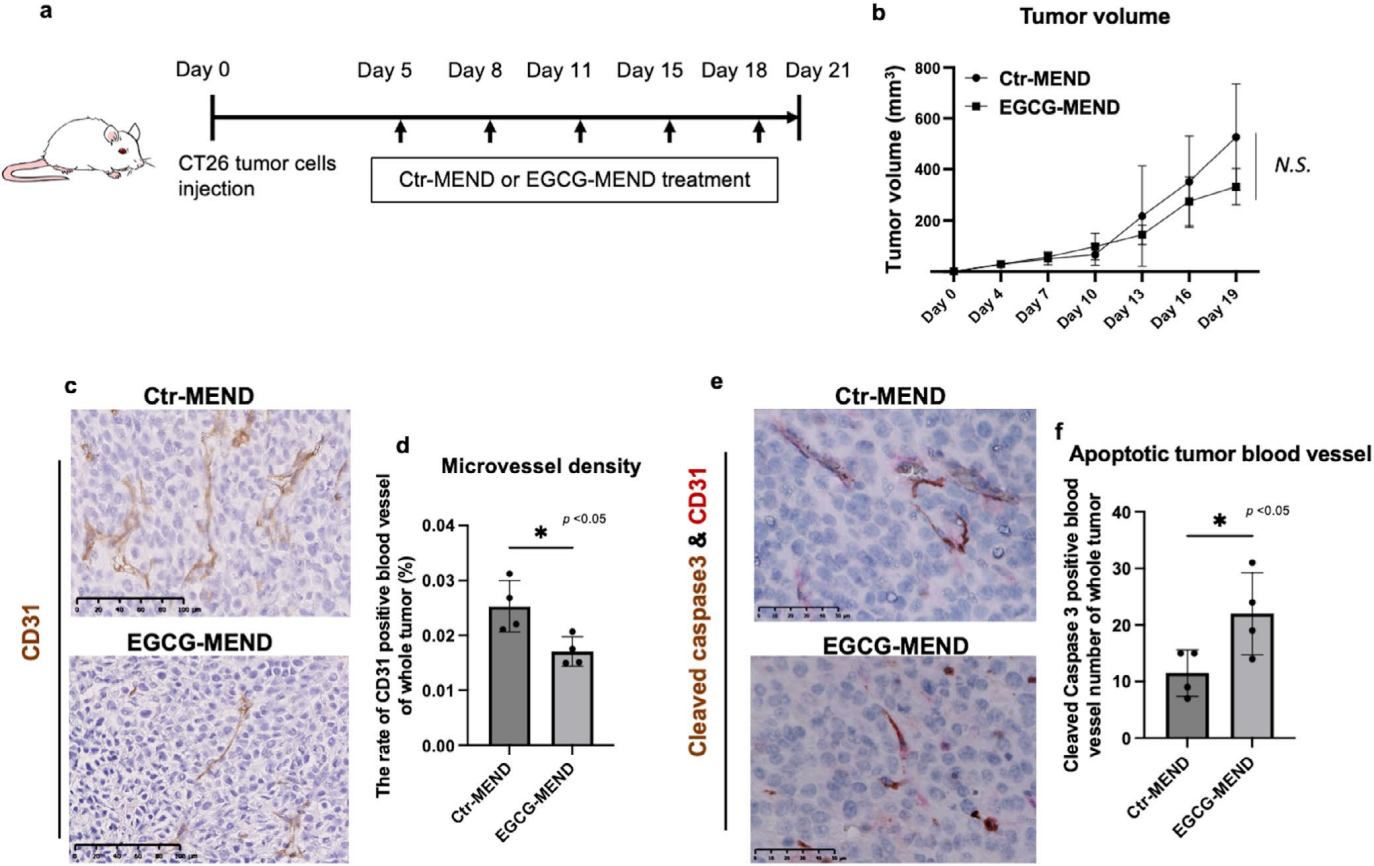
EGCG‐MEND exerts anti‐angiogenic effects. (a) Experimental design for the evaluation of the therapeutic efficacy of EGCG‐MEND. Ctr‐MEND or EGCG‐MEND was injected at the indicated time point (arrow). (b) Tumor growth curves. (c, d) Tumor blood vessels were visualized by CD31 immunostaining. Scale bar = 100 μM (c). The tumor MVD of the whole tumor was analyzed by quantifying the CD31‐stained area using ImageJ (d). (e, f) Necrotic tumor vessels were identified by immunohistochemical detection of vessels that were positive for both cleaved caspase‐3 (brown) and CD31 (red). Scale bar = 50 μM (e). Double‐positive vessels were counted in the whole tumor as necrotic tumor blood vessels (f). All data are presented as means ± SDs, *p < 0.05, **p < 0.01, N.S., not significant, two‐tailed unpaired Student's *t*‐test.

To evaluate the effect of EGCG on TECs in vivo, EGCG was encapsulated with cRGD‐MEND [47] to achieve targeted delivery of EGCG to TECs. Briefly, to deliver it specifically to TEC, we added cRGD because α V β 3 integrin, which is a cRGD receptor, is selectively expressed at high levels in TEC. YSK05 and other lipids were dissolved in a 90% tert‐butanol aqueous solution during MEND preparation, and an EGCG solution of 10 mg/mL was added to the lipid solution under vortex mixing. Subsequently, RGD modification was conducted by incubating YSK‐MEND with RGD‐PEG solution at 60°C. After tumor palpation, EGCG‐MEND (10 mg/kg/mice) was administered via a tail vein every 3 days in CT26 tumor‐bearing mice (Figure 3a). Tumor size changes indicated a trend of tumor growth inhibition in the EGCG‐MEND group (Figure 3b). Microvessel density (MVD) was analyzed to determine anti‐angiogenic effect of EGCG. In comparison with the Ctr‐MEND group, the EGCG‐MEND group exhibited a significant reduction in MVD (Figure 3c,d). In addition, cleaved caspase‐3 and CD31 co‐staining analysis demonstrated more than 1‐fold increased cleaved caspase‐3 and CD31 double‐positive blood vessels in the EGCG‐MEND group (Figure 3e,f). These findings indicate that EGCG‐targeted delivery to the tumor vasculature inhibits tumor angiogenesis.

### 3.4 EGCG‐MEND Exerts Anti‐Inflammatory and Antithrombotic Effects

**FIGURE 4:**
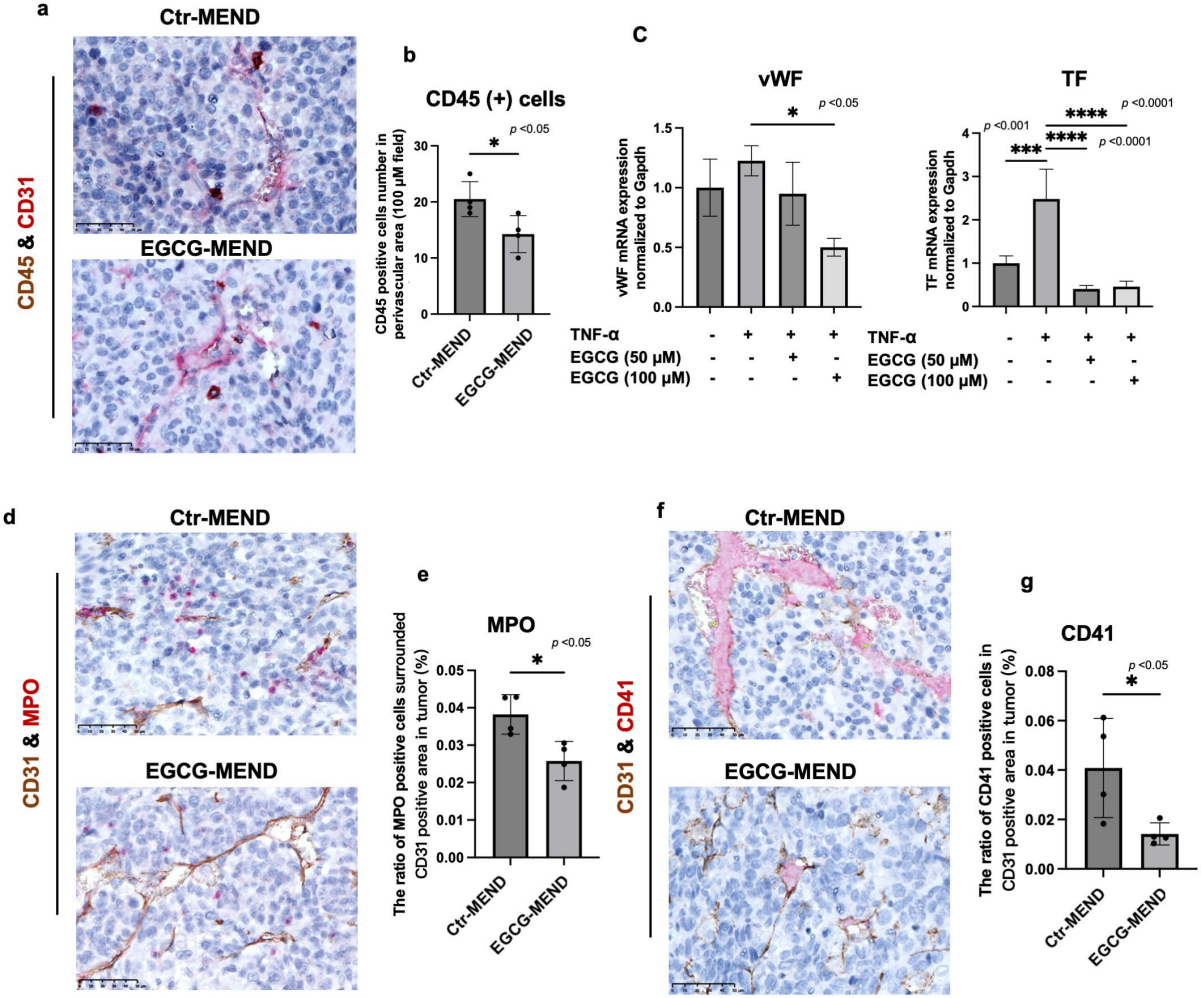
EGCG‐MEND exerts an anti‐inflammatory and antithrombotic effect. (a) Perivascular inflammation was detected by CD45 and CD31 immunohistochemistry. (b) CD45‐positive cells around CD31‐positive vessels were counted; five fields per tumor. Scale bar = 50 μm. (c) TECs were exposed to 10 ng/mL TNF‐α for 6 h and then treated with EGCG for 6 h, and the expression levels of vWF and TF were determined by RT‐qPCR. (d) Representative images of MPO staining in tumors. Scale bar = 50 μm. (e) Quantification of MPO‐positive cells around CD31‐positive vessels in the tumors. (f) Representative CD41 and CD31 immunostaining images in the Ctr‐MEND and EGCG‐MEND groups. Scale bar = 50 μm. (g) The CD41‐positive area in the CD31‐positive area of the whole tumor was analyzed using ImageJ. *p < 0.05 ****p < 0.0001, Student's *t*‐test (b,e,g); one‐way ANOVA (c).

During inflammation, vascular inflammation promotes leukocyte recruitment [48]. To analyze the anti‐inflammatory effect of EGCG on TECs, the mobilization of immune cells around the blood vessels in the tumors was first analyzed by double staining of CD31 and CD45. The number of CD45‐positive cells in perivascular areas was significantly reduced in the EGCG‐MEND group (Figure 4a,b). Because inflammatory factors such as IL‐1β, TNF‐α, and cell adhesion molecules that regulate extravasation of leukocytes were reported to mediate their recruitment to inflammation sites [49], the delivery of EGCG to tumor vasculature using RGD‐MEND may inhibit vascular inflammation with the inhibition of CD45‐positive leukocyte infiltration. Inflammatory cytokines, such as TNF‐α, induce the procoagulant phenotype of ECs with the upregulation of *TF* or *vWF* genes [50]. Then, the effect of EGCG on procoagulant phenotype in ECs with TNF‐α stimulation was analyzed in vitro. EGCG treatment significantly decreased vWF and TF expression levels in TECs. Specifically, EGCG reduced vWF gene expression by up to 50%, and TF expression decreased more than fivefold compared to TNF‐α stimulation (Figure 4c). These results suggest that the inhibition of inflammation by EGCG could reduce the risk of thrombosis. Tumor inflammation is reported not only to induce platelet activation to promote a procoagulant phenotype but also to stimulate neutrophils to release neutrophil extracellular traps (NETs), which is involved in thrombus formation [51]. Because MPO is a representative component of NETs, MPO was visualized by IHC. Interestingly, a decrease in MPO‐positive cells by EGCG treatment was found in perivascular area(Figure 4d,e). Then, thrombus formation in tumors with CD41 staining was analyzed to visualize platelet aggregation. A significant reduction of the CD41‐positive area in the CD31‐positive area was observed in the EGCG‐MEND group (Figure 4f,g). In our study, EGCG treatment reduced inflammatory factor levels in TEC (Figure 1c, TNF‐α, IL‐6, **p < 0.01; IL‐1β, *p < 0.05), with corresponding reductions in the thrombotic markers vWF and TF (Figure 4c, vWF, *p < 0.05; TF, ****p < 0.0001). Additionally, in vivo tests confirmed the association of reduced thrombosis in EGCG‐treated tumor models (Figure 4f,g, *p < 0.05). These data confirm that the ability of EGCG to inhibit vascular inflammation may contribute to its antithrombotic effects.

### 3.5 EGCG‐MEND Treatment Activates Tumor Immunity

**FIGURE 5:**
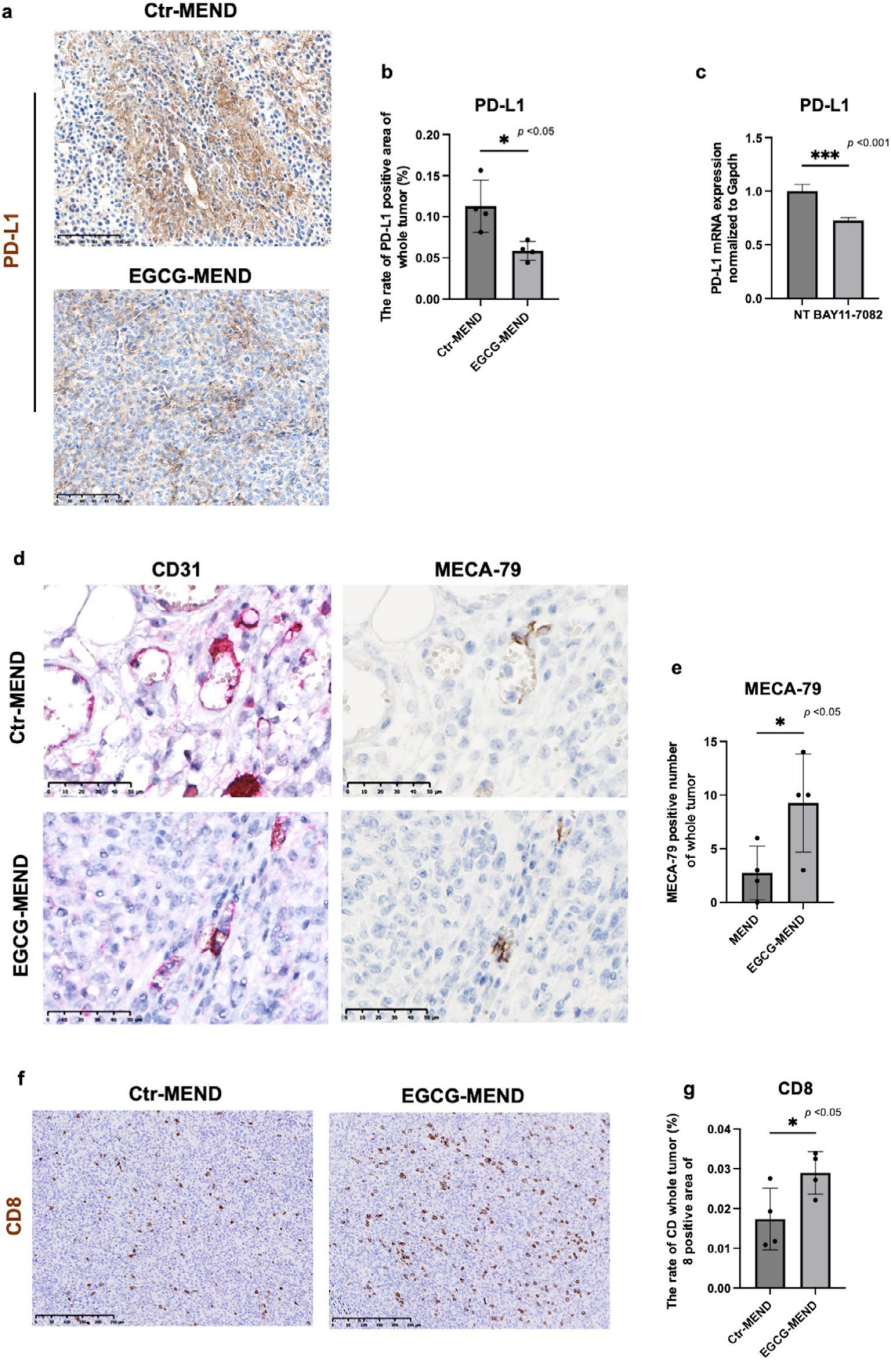
EGCG‐MEND treatment activates tumor immunity by suppressing inflammation. (a) Representative images of PD‐L1 immunostaining in the Ctr‐MEND and EGCG‐MEND groups. Scale bar = 100 μm. (b) Quantification of PD‐L1 staining areas was analyzed by ImageJ. (c) The PD‐L1 mRNA expression in TECs was determined by RT‐qPCR after treatment with the BAY11‐7082 for 24 h. (d) Representative CD31 and MECA‐79 immunostaining images in the Ctr‐MEND and EGCG‐MEND groups. Scale bar = 50 μm. (e) Quantification of MECA‐79 staining areas was analyzed by ImageJ. (f) Representative images of CD8α immunostaining in the Ctr‐MEND and EGCG‐MEND groups. Scale bar = 250 μm. (g) Quantification of CD8α‐stained areas was analyzed by ImageJ, *p < 0.05, Student's *t*‐test.

Recently, immune‐based therapies have developed and shown significant therapeutic effects on many tumors [52]. The expression level of PD‐L1, an immune checkpoint ligand, is closely related to the inflammatory state [53]. Moreover, the possible effect of EGCG on the regulation of the tumor immune microenvironment was investigated. In the IHC, PD‐L1 had significantly reduced expression in the EGCG‐MEND group (Figure 5a,b). PD‐L1 expression is upregulated through signaling pathways including NF‐κB [54]. The treatment of TECs with NF‐κB inhibitors significantly reduced the PD‐L1 expression levels (Figure 5c). Because the combination of anti‐angiogenic and anti‐PD‐L1 therapy induces high endothelial venule (HEV) and enhances infiltration of CD8^+^ T cells [55], we checked HEV formation in the tumors. The increase in the MECA‐79‐positive area in the EGCG‐MEND group showed that EGCG treatment induced HEV formation (Figure 5d,e). In addition, EGCG treatment increased the number of CD8^+^ T cells in the tumors (Figure 5f,g). These data suggest that EGCG may improve tumor immunity by suppressing inflammation.

## 4 Discussion

**FIGURE 6:**
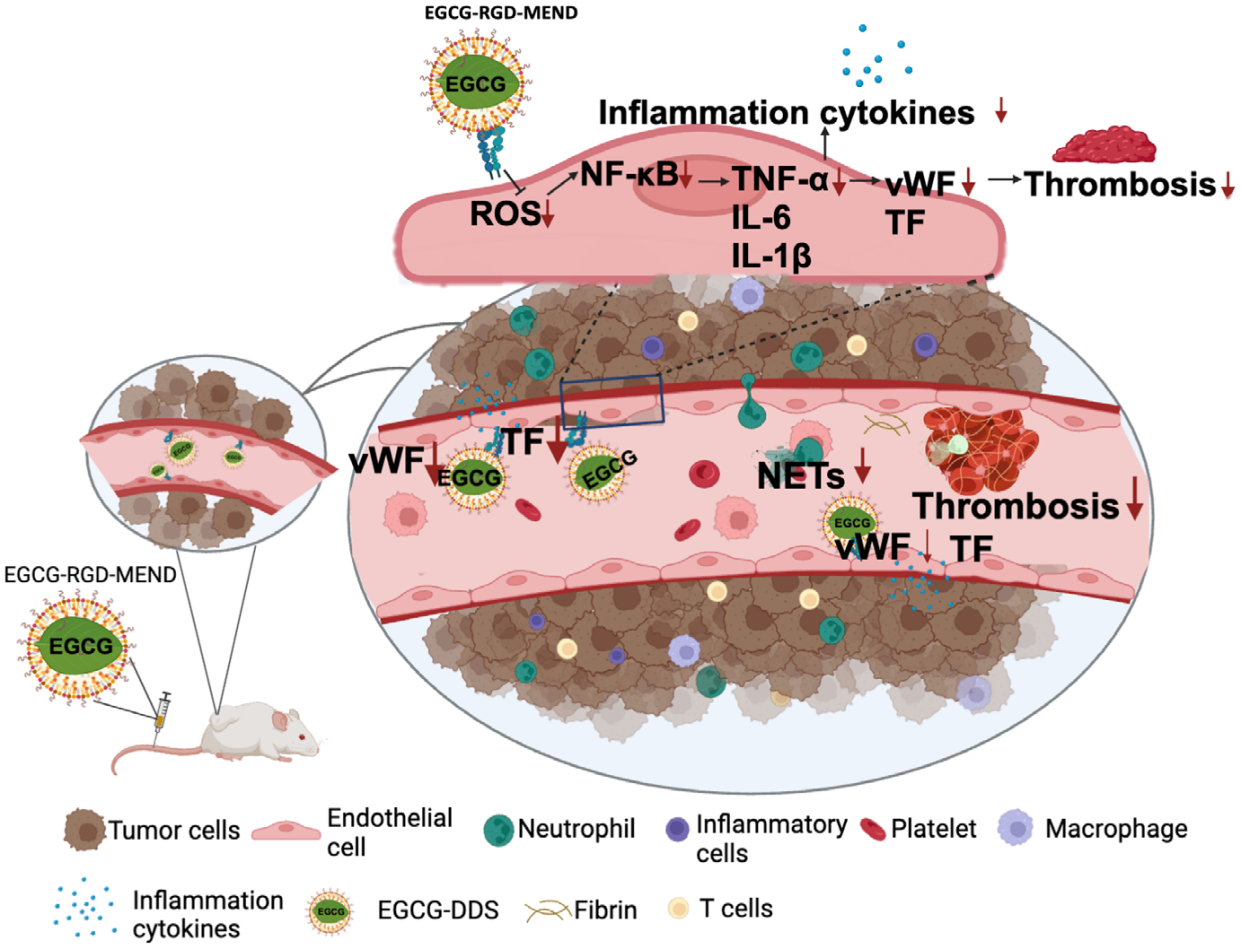
Schematic image of the mechanism by which EGCG inhibits inflammation and thrombosis in TECs. EGCG scavenges ROS in TECs and inhibits NF‐kB activation, thereby suppressing the expression of NF‐kB‐regulated inflammation‐related factors (TNF‐α, IL‐6, and IL‐1β). The expression of the vWF and TF gene is regulated by TNF‐α, and thus, EGCG inhibits thrombosis formation.

In this study, targeting TECs by EGCG‐MEND induced anti‐inflammatory effects by suppressing NF‐kB activation, thereby reducing the risk of thrombosis, a leading direct cause of death in patients with cancer (Figure 6) (Created with BioRender.com).

Chronic inflammation is observed in the tumor microenvironment, referred to as “wounds that do not heal” [56]. The proinflammatory mediators in the tumor microenvironment orchestrate crosstalk among cells to create a tumor‐promoting microenvironment including immunosuppression and angiogenesis. TECs lining the blood vessels of the tumor were also described as “activated” and “chronically inflamed” [57]. TECs release inflammatory mediators such as ILs and TNF‐α, which further trigger an inflammatory response that affects surrounding tumor and immune cells [58]. EGCG is a major catechin and a major bioactive compound in green tea leaves and is considered to play a crucial role in anticancer activities [59]. Because the present study showed that TECs have elevated levels of inflammatory factors, EGCG was expected to reduce the expression of inflammatory factors in TECs. Consistent with our previous reports [9], TECs showed a higher sensitivity to EGCG than NECs in terms of the inhibition of angiogenesis and anti‐inflammatory effects.

ROS are key signaling molecules in the progression of inflammation [60]. High levels of ROS were often associated with increased inflammatory signaling in tumors [61]. ROS promote the activation of proinflammatory cytokines through the activation of signaling pathways such as NF‐κB, COX‐2, and STAT3, and antioxidants can inhibit these processes [62]. ROS also induce various proinflammatory pathways in ECs, such as apoptosis signal‐regulated kinase 1 [63] and mitogen‐activated protein kinase (MAPK) [64]. NF‐κB activity is regulated by ROS [65]. NF‐κB plays a key role in tumorigenesis and progression and participates in the regulation of proinflammatory genes to produce proinflammatory cytokines such as TNF‐α, IL‐1β, and IL‐6 [66]. EGCG was reported to inhibit the phosphorylation of NF‐kB and cause anti‐inflammation effect on TNF‐α stimulated human umbilical vein endothelial (HUVECs) cells [67]. Consistent with our results, EGCG treatment inhibited the phosphorylation of NF‐κB and its downstream inflammatory cytokine expression in TECs (Figure 2e). In addition, because EGCG possesses multiple anti‐inflammatory pathways such as the regulation of MAPK [68] and adhesion molecules [69], our results may also show a role for other pathways, which needs to be further tested in subsequent studies.

EGCG has low bioavailability and is influenced by various factors within the body, such as pH and temperature [70]. EGCG in combination with drug delivery systems is currently being extensively explored to improve stability and effectiveness [71]. To target TECs with EGCG in vivo more efficiently, the nanodrug delivery system cRGD‐MEND was established, where αvβ3 integrins are selectively expressed at high levels in TECs as receptors for RGD [30]. Previously, we demonstrated selective delivery of RGD‐MEND encapsulating siRNA into the tumor vasculature [32]. With TEC‐targeted RGD‐MEND, silencing of VEGFR2, one of the important VEGF‐A receptors, induced anti‐angiogenic and antitumor effects [31]. Our other study provided the inhibitory mechanism of EGCG on tumor angiogenesis, including the inhibition of TEC migration toward VEGF, inhibition of EGCG‐mediated Akt phosphorylation in TECs, and the suppression of VEGF‐induced mobilization of CD133+/VEGFR2+ cells into the peripheral circulation [9]. Ultimately, these findings contribute to the anti‐angiogenic activity of EGCG. After EGCG was delivered to the tumor vessels, angiogenesis was significantly reduced (Figure 3c,d), and apoptotic cells in the tumor blood vessels were confirmed (Figure 3e,f). A previous study reported that EGCG treatment promoted tumor vascular normalization, including a reduction in MVD and type IV collagen expression, along with a transient increase in pericyte coverage and blood perfusion [72]. This effect is probably achieved by targeting the VEGF pathway [73]. However, no significant difference in pericyte coverage was found between the two groups (data not shown). This may be because of the specific targeting of DDS on TECs, whereas VEGF is mainly secreted by tumor and stromal cells [74]. EGCG alone has exhibited significant anti‐angiogenic effects, but a direct comparison with the RGD‐MEND encapsulation approach warrants further investigation. The current data are insufficient to identify the superiority of EGCG treatment over RGD‐MEND encapsulation for vessel normalization. Future studies should aim to directly compare these approaches to assess their relative efficacy in vessel normalization.

Leukocytes are commonly located in the perivascular periphery of tumor vessels [17]. Indeed, in our mouse model, CD45‐positive inflammatory cells were detected around the blood vessels in the tumors (Figure 4a,b). Inflammatory states upregulated adhesion molecules on TECs [56], promoting the adhesion of immune cells such as leukocytes to the vascular endothelium. This adhesion leads to the release of inflammatory mediators, sustaining the inflammatory process [75]. TECs overexpress proinflammatory molecules to mobilize leukocytes. Our results demonstrate a significant reduction in the number of inflammatory cells around the vasculature after EGCG treatment. Under inflammatory stimulation, numerous immune cells, including neutrophils, are recruited to the inflammatory site [76]. Neutrophils play various roles during inflammation, including phagocytosis of microorganisms, ROS production, and NETs formation [77]. Our study revealed that EGCG scavenged ROS levels in TECs (Figure 2a,b). Therefore, we revealed how EGCG affected specific inflammation markers and thrombosis, including CD45‐positive cells, vWF, TF, MPO‐positive cells, and CD41‐positive areas. Previous studies have demonstrated that reducing ROS inhibits NF‐κB and MAPK pathways, thereby decreasing inflammation [78]. This is consistent with our observations of reduced inflammatory marker expression and thrombosis in TECs. Moreover, our results provide the first direct evidence of EGCG's effect on thrombus formation within tumors, emphasizing its potential as a novel therapeutic strategy for addressing tumor‐associated thrombosis.

Tumor immune escape is a major strategy for cancer survival and progression [79]. The interaction between the programmed cell death 1 (PD‐1) receptor on T cells and PD‐L1 induces T‐cell exhaustion [80]. Immunotherapy targeting immune checkpoints has shown tremendous potential in cancer treatment [52]. PD‐L1 expression is upregulated through signaling pathways including NF‐κB, MAPK, and JAK–STAT [81]. Our results indicated a reduction in PD‐L1 expression in the tumors of the EGCG‐MEND group compared to Ctr‐MEND group (Figure 5a,b, *p < 0.05), which may be mediated by the inhibition of NF‐kB activity by EGCG. The combination of anti‐PD‐L1 and anti‐angiogenic therapy was reported to induce HEV formation in tumors [55]. Present data indicated that EGCG inhibited PD‐L1 expression in tumors and possessed an anti‐angiogenic effect. Expectedly, more HEV formation was observed in the EGCG‐MEND group compared with the control group (Figure 5d,e, *p < 0.05). In addition, the EGCG‐MEND‐treated tumors had more T‐cell infiltration. This could be because the reduction of PD‐L1 expression relieves the inhibition of T cells, allowing T cells to recognize and attack tumor cells more effectively [82]. Moreover, HEVs may contribute to the recruitment of immune cells including T cells into the tumor tissues [83].

In this study, TEC‐targeted DDS was employed to verify the effect of EGCG on TECs. However, targeting TEC by EGCG‐MEND leads to changes in PD‐L1 expression throughout the entire tumor, rather than just around the blood vessels. Further investigations are required to address whether the downregulation of inflammatory cytokines in TEC leads to decreased PD‐L1 expression in tumor cells. EGCG has exhibited therapeutic effects on cancer cells [84, 85], ECs [9], immune cells [86], cancer‐associated fibroblasts [87], and tumor‐associated macrophages [88], the antithrombotic effects of EGCG in tumors, particularly on the thrombogenic phenotype of TECs, have not been extensively explored in the literature. Prior studies have primarily focused on EGCG's anti‐inflammatory [24] and anticancer properties [23], with limited emphasis on its role in modulating thrombogenic responses in the tumor microenvironment. Our study is the first study providing evidence of the effect of EGCG on thrombogenic phenotype in TEC, to the best of our knowledge, offering novel insights and therapeutic avenues for EGCG as a cancer associated thrombosis treatment modality.

In conclusion, our study showed that targeted delivery of EGCG in tumor blood vessels may improve the patient's prognosis by exerting anti‐inflammatory and antithrombotic effects and improving antitumor immunity.

## Author Contributions

**Zi Jia:** formal analysis (lead), methodology (equal), validation (equal), visualization (lead), writing – original draft (lead). **Nako Maishi:** conceptualization (supporting), funding acquisition (equal), methodology (equal), resources (equal), supervision (equal), writing – review and editing (lead). **Hideki Takekawa:** formal analysis (equal), methodology (supporting), validation (equal), visualization (supporting). **Aya Yanagawa Matsuda:** supervision (equal), validation (supporting), writing – review and editing (equal). **Taisei Nakade:** methodology (supporting), resources (supporting). **Takashi Nakamura:** resources (equal), supervision (supporting). **Hideyoshi Harashima:** resources (supporting), supervision (supporting). **Yasuhiro Hida:** funding acquisition (equal), supervision (supporting). **Kyoko Hida:** conceptualization (lead), funding acquisition (lead), project administration (lead), supervision (lead), writing – review and editing (lead).

## Ethics Statement

Approval of the research protocol by an Institutional Reviewer Board: N/A. Animal Studies: All animal experiments were approved by the animal research authorities of Hokkaido University (approval number: 20‐0113). The authors followed the Animal Research: Reporting of In Vivo Experiments (ARRIVE) guidelines for animal studies.

## Consent

The authors have nothing to report.

## Conflicts of Interest

The authors declare no conflicts of interest.

## Supporting information


**Appendix S1.** Detail descriptions of the methods.

Table S1.

Table S2.

## Data Availability

The datasets used during the current study are available from the corresponding author on reasonable request.
