# Supplementary material for: Targeting Tumor Endothelial Cells by EGCG Using Specific Liposome Delivery System Inhibits Vascular Inflammation and Thrombosis
Source: Cancer Med. 2024 Dec 4;13(23):e70462. doi: 10.1002/cam4.70462 (PMC11615514; doi:10.1002/cam4.70462)
Supplement: Supplementary file 1 — Appendix S1. Detail descriptions of the methods. [file CAM4-13-e70462-s002.docx]

**Targeting tumor endothelial cells by EGCG – liposome delivery system inhibits vascular inflammation and thrombosis**

Zi Jia^1a^, Nako Maishi^1a^, Hideki Takekawa^1^, Aya Matsuda^1^, Taisei Nakade^2^, Takashi Nakamura^2^, Hideyoshi Harashima^2^, Yasuhiro Hida^3^, Kyoko Hida^1*^

**Supplementary Materials and Methods**

***Immunohistochemistry (IHC)***

Tumor tissues were dissected from mice, halved, processed, and embedded in paraffin. Then, 4-µm sections were prepared for staining with hematoxylin and eosin (HE), anti-CD31 (28364, Abcam), anti-cleaved caspase-3 (9664-S, Cell Signaling), anti-CD45 (103112, Biolegend), anti-myeloperoxidase (MPO) (9535, Abcam), anti-CD41 (134141, Abcam), anti-mouse PD-L1/B7-H1 (Bio-techneAF1019), anti-CD8a (100702, Biolegend) antibodies. Immunoreaction was visualized with HRP-linked secondary antibody (Dako) and DAB substrate (Dako). The Vulcan Fast Red Chromogen Kit 2 (Biocare Medical, BRR805AS) was used for alkaline phosphatase (AP) color development using an AP-linked secondary antibody (Dako). Hematoxylin (Wako) was used to counterstain sections.

***Evaluation of IHC staining***

CD31-, programmed death-ligand 1 (PD-L1)-, and CD8a-positive staining areas were quantified using ImageJ. The percentages of the positive areas were calculated to find the total area. Five fields per sample (CD31) or the positive areas in the entire tumor area (PD-L1 and CD8) were quantified, and their values were averaged to obtain one value per sample. Each group consisted of four mice. For the quantitative analyses of caspase-3, CD45, CD41, and MPO in the perivascular region, measurements were performed by co-staining with CD31. Five hotspots (blood vessel-rich areas) were selected by measuring the CD31-positive area [32], and the percentage of the total number of cleaved caspase-3, CD45-, CD41- or MPO-positive cells surrounding blood vessels in the hotspot region were calculated.
