# Supplementary material for: Targeting Tumor Endothelial Cells by EGCG Using Specific Liposome Delivery System Inhibits Vascular Inflammation and Thrombosis
Source: Cancer Med. 2024 Dec 4;13(23):e70462. doi: 10.1002/cam4.70462 (PMC11615514; doi:10.1002/cam4.70462)
Supplement: Supplementary file 2 — Table S1. [file CAM4-13-e70462-s001.docx]

**Supplementary Table S1 List of primers for PCR analysis.**

| **genes** | **Sequences** |
| --- | --- |
| ***Gapdh*** | Forward 5’GGGTGGTGGACCTCATGGCCTACAT3’ |
|  | Reverse 5’CGAGTTGGGATAGGGCCTCTCTTGC3’ |
| ***Il-6*** | Forward 5’AGCTGGAGTCACAGAAGGAGTGGC3’ |
|  | Reverse 5’GGCATAACGCACTAGGTTTGCCGAG3’ |
| ***Il-1β*** | Forward 5’TGCCACCTTTTGACAGTGATGAG3’ |
|  | Reverse 5’TGTGCTGCTGCGAGATTTGAAG3’ |
| ***Il-8*** | Forward 5’AGAGATACCGCCACGTTCTGAC3’ |
|  | Reverse 5’GGACACAGTGTTCTTGCCTTGG3’ |
| ***Tnf-α*** | Forward 5’ GCCTGTAGCCCACGTCGTACAAAC3’ |
|  | Reverse 5’ACAAGGTACAACCCATCGGCTGGC3’ |
| ***vWF*** | Forward 5’ CGGCTTGCACCATTCAGC3’ |
|  | Reverse 5’ CCATCCTGGAGCGTCTCATC3 |
| ***TF*** | Forward 5’ TACCGAGACACAAACCTCGGAC3’ |
|  | Reverse 5’ TTTTCTTTCCCGTGCTTGAGCC3 |
| ***PD-L1*** | Forward 5’ TGCGGACTACAAGCGAATCACG3’ |
|  | Reverse 5’ CTCAGCTTCTGGATAACCCTCG3 |
