# Supplementary material for: Targeting Tumor Endothelial Cells by EGCG Using Specific Liposome Delivery System Inhibits Vascular Inflammation and Thrombosis
Source: Cancer Med. 2024 Dec 4;13(23):e70462. doi: 10.1002/cam4.70462 (PMC11615514; doi:10.1002/cam4.70462)
Supplement: Supplementary file 3 — Table S2. [file CAM4-13-e70462-s003.docx]

| **EGCG RGD-MEND** | **EGCG conc. (µg/mL)** | **Recovery rate** |
| --- | --- | --- |
|  | **1048.15** | **108.1014182** |

| **MEND** | diameter **(nm)** | ζ-potential (mV) | **PDI** |
| --- | --- | --- | --- |
| **EGCG RGD-MEND** | **236.5** | **-10.1** | **0.073** |
| **Empty RGD-MEND** | **154.6** | **-3.29** | **0.072** |
